# Administration of aerosolized SARS-CoV-2 to K18-hACE2 mice uncouples respiratory infection from fatal neuroinvasion

**DOI:** 10.1126/sciimmunol.abl9929

**Published:** 2021-11-23

**Authors:** Valeria Fumagalli, Micol Ravà, Davide Marotta, Pietro Di Lucia, Chiara Laura, Eleonora Sala, Marta Grillo, Elisa Bono, Leonardo Giustini, Chiara Perucchini, Marta Mainetti, Alessandro Sessa, José M. Garcia-Manteiga, Lorena Donnici, Lara Manganaro, Serena Delbue, Vania Broccoli, Raffaele De Francesco, Patrizia D’Adamo, Mirela Kuka, Luca G. Guidotti, Matteo Iannacone

**Affiliations:** ^1^ Division of Immunology, Transplantation, and Infectious Diseases, IRCCS San Raffaele Scientific Institute, 20132 Milan, Italy.; ^2^ Vita-Salute San Raffaele University, 20132 Milan, Italy.; ^3^ Center for Omics Sciences, IRCCS San Raffaele Scientific Institute, 20132 Milan, Italy.; ^4^ Division of Neuroscience, IRCCS San Raffaele Scientific Institute, 20132 Milan, Italy.; ^5^ INGM - Istituto Nazionale di Genetica Molecolare “Romeo ed Erica Invernizzi”, Milan, Italy; ^6^ Department of Biomedical, Surgical and Dental Sciences, University of Milan, Italy; ^7^ National Research Council of Italy, Institute of Neuroscience; ^8^ Department of Pharmacological and Biomolecular Sciences (DiSFeB), University of Milan, Italy; ^9^ Center of Advanced Services for in-vivo testing – Animal behavior Facility, IRCCS San Raffaele Scientific Institute, 20132 Milan, Italy.; ^10^ Experimental Imaging Centre, IRCCS San Raffaele Scientific Institute, 20132 Milan, Italy.

## Abstract

The development of a tractable small animal model faithfully reproducing human COVID-19 pathogenesis would arguably meet a pressing need in biomedical research. Thus far, most investigators have used transgenic mice expressing the human ACE2 in epithelial cells (K18-hACE2 transgenic mice) that are intranasally instilled with a liquid SARS-CoV-2 suspension under deep anesthesia. Unfortunately, this experimental approach results in disproportionate high CNS infection leading to fatal encephalitis, which is rarely observed in humans and severely limits this model’s usefulness. Here, we describe the use of an inhalation tower system that allows exposure of unanesthetized mice to aerosolized virus under controlled conditions. Aerosol exposure of K18-hACE2 transgenic mice to SARS-CoV-2 resulted in robust viral replication in the respiratory tract, anosmia, and airway obstruction, but did not lead to fatal viral neuroinvasion. When compared to intranasal inoculation, aerosol infection resulted in a more pronounced lung pathology including increased immune infiltration, fibrin deposition and a transcriptional signature comparable to that observed in SARS-CoV-2-infected patients. This model may prove useful for studies of viral transmission, disease pathogenesis (including long-term consequences of SARS-CoV-2 infection) and therapeutic interventions.

## Teaser

Aerosol exposure of K18-hACE2 mice to SARS-CoV-2 leads to respiratory infection and anosmia but not to fatal neuroinvasion.

## INTRODUCTION

The coronavirus disease 2019 (COVID-19) pandemic is caused by the recently identified β-coronavirus severe acute respiratory syndrome coronavirus 2 (SARS-CoV-2) (*1*, *2*). Disease severity is variable, ranging from asymptomatic infection to multi-organ failure and death. Although SARS-CoV-2 primarily targets the respiratory system, some patients with COVID-19 can also exhibit extrarespiratory symptoms, including neurological manifestations such as loss of smell (anosmia) and taste (ageusia), headache, fatigue, memory impairment, vomiting, gait disorders and impaired consciousness (*3*–*6*). SARS-CoV-2 can infect neurons in human brain organoids (*7*, *8*) and a few studies reported the presence of SARS-CoV-2 in olfactory sensory neurons (OSNs) and deeper areas within the central nervous system (CNS) in fatal COVID-19 cases (*8*–*12*). However, the neurotropism of SARS-CoV-2 and a direct role of CNS infection in the pathogenesis of neurological manifestations remains highly debated.

Despite the availability of effective vaccines against SARS-CoV-2, we still know little about COVID-19 pathogenesis. The availability of tractable animal models to mechanistically dissect virological, immunological and pathogenetic aspects of the infection with SARS-CoV-2 and future human coronaviruses would provide major benefit. Wild-type laboratory mice are poorly susceptible to SARS-CoV-2 infection because the mouse angiotensin-converting enzyme (ACE) 2 does not act as a cellular receptor for the virus (*13*). Several transgenic mouse lineages expressing the human version of the SARS-CoV-2 receptor (hACE2) support viral replication and recapitulate certain clinical characteristics of the human infection (*13*). The most widely used model is the K18-hACE2 transgenic mouse (*14*), which expresses hACE2 predominantly in epithelial cells under the control of the cytokeratin 18 (*KRT18*) promoter (*15*). K18-hACE2 mice are typically infected by intranasally instilling liquid suspensions of SARS-CoV-2 under deep anesthesia. This results in disproportionate high CNS infection leading to fatal encephalitis (*16*–*20*), which rarely occurs in patients with COVID-19. Such viral neuroinvasion severely limits the usefulness of these mouse models, hampering studies on disease pathogenesis (including long-term consequences of SARS-CoV-2 infection) as well as on drug discovery. Here we report the generation and characterization of an alternative COVID-19 platform, based on controlled exposure of K18-hACE2 transgenic mice to aerosolized SARS-CoV-2.

## RESULTS

### Intranasal inoculation, but not aerosol exposure, of SARS-CoV-2 leads to fatal neuroinvasion in K18-hACE2 transgenic mice.

**Fig. 1.**
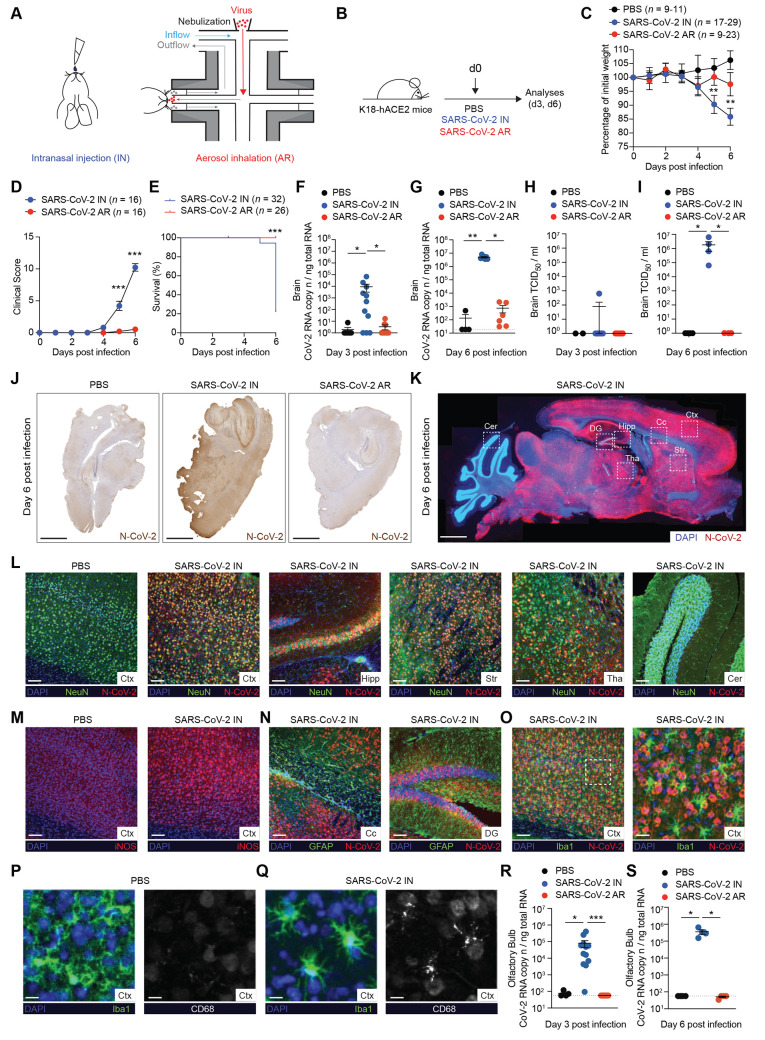
**Intranasal inoculation, but not aerosol exposure, of SARS-CoV-2 leads to fatal neuroinvasion in K18-hACE2 transgenic mice. (A)** Illustration of the two modalities used to infect K18-hACE2 mice with SARS-CoV-2. On the left, intranasal injection (IN) is shown. On the right, representation of an unanesthetized mouse placed in the nose-only Allay restrainer on the inhalation chamber is shown. In red, aerosolized virus with a particle size of ~4 μm; in light-blue, primary flow set to 0,5 L/min/port; in grey, mouse breathing outflow (see Methods). **(B)** Schematic representation of the experimental setup. K18-hACE2 mice were infected with a target dose of 1 × 10^5^ TCID_50_ of SARS-CoV-2 through intranasal (IN) administration or through aerosol (AR) exposure. Lung, brain, nasal turbinates, olfactory bulbs, and blood were collected and analyzed 3 days and 6 days post infection. **(C)** Mouse body weight was monitored daily for up to 6 days and is expressed as the percentage of weight relative to the initial weight on day 0. Statistical significance of comparison between IN- (*n* = 17-29, blue dots) and AR-infected mice (*n* = 9-23, red dots) is shown. Control mice treated with PBS are also shown (*n* = 9-11, black dots). Data are represented as mean ± SEM. *n* indicate a range of mice, some of them have been dropped out from the analysis to be sacrificed for planned experimental end-points. **(D)** Clinical score was assessed evaluating the piloerection (0-3), posture (0-3), activity level (0-3), eye closure (0-3) and breathing (0-3) (see Methods). Statistical significance of comparison between IN- (*n* = 16, blue dots) and AR-infected mice (*n* = 16, red dots) is shown. PBS-treated control mice did not exhibit any clinical signs. **(E)** Survival curve of IN- (*n* = 32, blue dots) and AR-infected mice (*n* = 26, red dots). **(F, G)** Quantification of SARS-CoV-2 RNA in the brain of IN- (*n* = 7-11, blue dots) and AR- (*n* = 6-10, red dots) infected mice as well as of PBS-treated control mice (*n* = 4, black dots) measured 3 days (**F**) and 6 days (**G**) post infection. RNA values are expressed as copy number per ng of total RNA and the limit of detection is indicated as a dotted line. **(H, I)** Viral titers in the brain were determined 3 (**H**) and 6 days (**I**) after infection by median tissue culture infectious dose (TCID_50_). PBS-treated control mice: *n* = 2-3, black dots; IN-infected mice: *n* = 4, blue dots; AR-infected mice: *n* = 4, red dots. **(J)** Representative immunohistochemical micrographs of sagittal brain sections from PBS-treated control mice (left), IN- (middle) and AR-infected mice (right) 6 days post infection. N-SARS-CoV-2 positive cells are depicted in brown. Scale bars, 1 mm. **(K)** Representative confocal immunofluorescence staining for N-CoV-2 (red) in sagittal brain sections of IN-infected mice. Cell nuclei are depicted in blue. White boxes indicate different brain areas: cerebellum (Cer); dentate gyrus (DG); hippocampus (Hipp); corpus callosum (Cc); cerebral cortex (Ctx); thalamus (Tha); striatum (Str). Scale bar, 1 mm. **(L)** Representative confocal immunofluorescence micrographs of sagittal brain sections from PBS-treated control mice (first panel) and IN-infected mice 6 days post infection. N-CoV-2 is depicted in red, NeuN neural marker in green and cell nuclei in blue. Fields of cerebral cortex (Ctx), hippocampus (Hipp), striatum (Str), thalamus (Tha) and cerebellum (Cer) are shown. Scale bars, 100 μm. **(M)** Representative confocal immunofluorescence micrographs of cerebral cortex (Ctx) in PBS-treated control mice (left panel) and IN-infected mice (right panel) 6 days post infection. iNOS^+^ cells are depicted in red and cell nuclei in blue. Scale bars, 100 μm. **(N)** Representative confocal immunofluorescence micrographs of two areas of the brain from IN-infected mice 6 days post infection. Corpus callosum (Cc) of the cerebral cortex, left panel, and dentate gyrus (DG), right panel. N-CoV-2 is depicted in red, GFAP astroglial marker in green and cell nuclei in blue. Scale bars, 100 μm. **(O)** Representative confocal immunofluorescence micrographs of the cerebral cortex (Ctx) of IN-infected mice at 6 days post infection. N-CoV-2 is depicted in red, Iba1 microglial marker in green and cell nuclei in blue. White box indicates the magnification represented in the right panel. Scale bars represent 100 μm (image) and 25 μm (magnification). **(P, Q)** Representative confocal immunofluorescence micrographs of the cerebral cortex (Ctx) of PBS-treated control mice (**P**) and IN-infected mice (**Q**) 6 days post infection. Left panels show Iba1 microglial marker in green and cell nuclei in blue; right panel shows CD68 marker of microglial activation in white. Scale bars, 10 μm. **(R, S)** Quantification of SARS-CoV-2 RNA in the olfactory bulbs of IN- (*n* = 4-12, blue dots) and AR- (*n* = 4-12, red dots) infected mice as well as of PBS-treated control mice (*n* = 2-4, black dots) measured 3 days (**R**) and 6 days (**S**) post infection. RNA values are expressed as copy number per ng of total RNA and the limit of detection is indicated as a dotted line. Data are expressed as mean ± SEM. Data in (C-I, R, S) are pooled from 2 independent experiments per time point. * p-value < 0.05, ** p-value < 0.01, *** p-value < 0.001; two-way ANOVA followed by Sidak’s multiple comparison test (**C, D,** comparison between blue and red dots for each time point); Log-rank (Mantel-Cox) test (**E**); Kruskal-Wallis test (**F**-**I, R-S**).

SARS-CoV-2 is mainly transmitted from person to person via respiratory droplets (*21*). In an attempt to mimic this transmission route, we made use of a nose-only inhalation tower system that allows to expose unanesthetized mice to aerosolized virus under controlled pressure, temperature, and humidity conditions (see **Figure S1A-C** and Methods). Animals were located inside a restraint with a neck clip positioned between the base of the skull and the shoulders, thus avoiding thorax compression, keeping the airways completely unobstructed and allowing for spontaneous breathing through the nose. K18-hACE2 transgenic mice were infected with a target dose of 1 × 10^5^ tissue culture infectious dose 50 (TCID_50_) of SARS-CoV-2 either through intranasal administration with 25 μl of diluted virus (IN) or through a 20 to 30 min exposure to aerosolized virus (AR) (**Fig. 1A-B**, see Methods). Pulmonary function was measured during aerosol exposure using plethysmography. Frequency, tidal volume, minute volume and accumulated volume of SARS-CoV-2-exposed mice were comparable to PBS-exposed mice (**Figure S1D**).

As expected (*19*, *20*), IN-infected animals exhibited significant body weight loss and a severe clinical score (see Methods for details), so that, by day 6 post infection (p.i.), ~ 80% of them had died and the remaining ones appeared lethargic (**Fig. 1C-E****, S2**). By contrast, AR-infected mice maintained stable body weight, and did not show any signs of disease nor mortality, including at 20 days p.i. (**Fig. 1C-E** and **Figure S2, S3**). The severe disease observed in IN-infected K18-hACE2 transgenic mice was associated with the detection of high viral RNA titers and infectious virus in the brain (**Fig. 1F****- I**). By contrast, neither SARS-CoV-2 RNA nor infectious virus were detected in the brain of mice exposed to aerosolized virus (**Fig. 1F-I**). Immunohistochemical and immunofluorescence staining confirmed the presence of the SARS-CoV-2 nucleoprotein in the brain of IN-infected, but not AR-infected, mice (**Fig. 1J, K**). Specifically, diffuse staining for SARS-CoV-2 nucleoprotein was detected throughout the cerebrum with comparable staining in the different brain areas with the notable exception of the cerebellum where most of its cells stained negative for viral antigens (**Fig. 1K**). Neurons were by far the most infected brain cells as shown by the co-staining of the SARS-CoV-2 nucleoprotein with the pan-neuronal marker NeuN (∼90% double positive cells, **Fig. 1L** and **Figure S4A**). Nitric oxide has been implicated as a contributor to the host's innate defense against viral infections including those affecting the CNS (*22*). Accordingly, neurons in infected brains strongly up-regulated iNOS (*22*), which was undetectable in neuronal cells from control, uninfected mice (**Fig. 1M**). By contrast, only a minor fraction of astrocytes (∼2%) and microglia (∼4%) stained positive for the SARS-CoV-2 nucleoprotein (**Fig. 1N, O** and **Figure S4B, C**). Of note, Iba1^+^ myeloid cells in SARS-CoV-2-infected brains were activated as revealed by the characteristic morphology (swollen processes with reduced ramifications) and CD68 positivity (**Fig. 1P, Q**). Consistent with the data on the recovery of infectious virus, viral RNA, and viral antigens, we found a significant immune cell recruitment (particularly of T cells, B cells, monocytes, and eosinophils) in the brains of IN-infected, but not AR-infected mice (**Figure S4D, E**). Together, these results demonstrate a profound viral neuroinvasion which correlates with the severe health deterioration in IN-infected mice. The high viral load and widespread viral distribution in the brain of IN-infected mice contrasts with the occasional localized detection of SARS-CoV-2 in the olfactory bulbs and/or the medulla of fatal COVID-19 cases (*10*, *23*–*27*) and caution against utilizing this model to investigate the neurological complications of SARS-CoV-2 infection in humans.

We next investigated the potential SARS-CoV-2 entry portals to the central nervous system (CNS) in IN-infected K18-hACE2 transgenic mice. One possibility is that the virus gains access to the CNS via the blood-brain barrier, which implies a viremic phase. However, no SARS-CoV-2 RNA was ever detected in the sera of infected mice (**Figure S4F, G**), consistent with earlier reports (*18*–*20*). Alternatively, SARS-CoV-2 could enter the CNS by retrograde axonal transport upon olfactory sensory neuron infection. Indeed, and in line with previous studies (*18*–*20*), viral RNA and viral antigens were detected in the olfactory bulb of IN-infected, but not AR-infected, mice (**Fig. 1R, S** and **Figure S5**). Overall, the data indicate that IN, but not AR, infection of K18-hACE2 transgenic mice with SARS-CoV-2 results in lethal neuroinvasion likely via retrograde axonal transport after olfactory sensory neuron infection.

### Aerosol exposure of K18-hACE2 transgenic mice to SARS-CoV-2 leads to efficient respiratory infection, anosmia, and fibrin deposition in the lung.

**Fig. 2.**
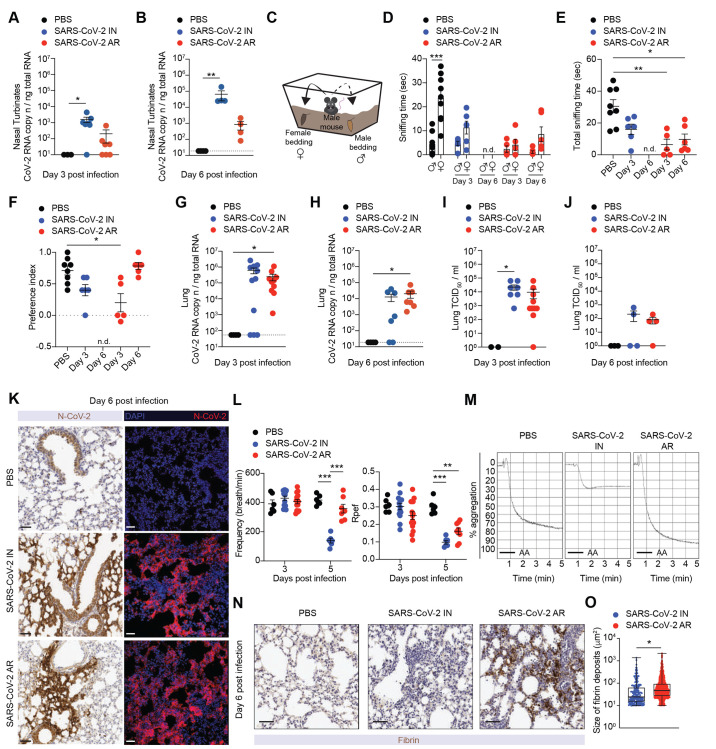
Aerosol exposure of K18-hACE2 transgenic mice to SARS-CoV-2 leads to efficient respiratory infection, anosmia, and fibrin deposition in the lung. **(A-B)** Quantification of SARS-CoV-2 RNA in the nasal turbinates of PBS-treated control mice (*n* = 3, black dots) and of IN- (*n* = 4-7, blue dots) and AR- (*n* = 4-7, red dots) infected mice 3 days (**A**) and 6 days (**B**) post infection. RNA values are expressed as copy number per ng of total RNA and the limit of detection is indicated as a dotted line. **(C)** Illustration showing social scent-discrimination test. Male mice were free to investigate for 5 min two different tubes containing their own cage bedding or female cage bedding placed at two opposite corners of a clean cage. **(D, E)** Time that males spent sniffing their own male scent or female scent (**D**) and the sum of the two times (**E**) is expressed as time sniffing (seconds). Analyses were performed 3 or 6 days post IN- (*n* = 3-6, blue dots) or AR-infection (*n* = 3-5, red dots). As control, PBS-treated mice are shown (*n* = 8, black dots). In D, comparison between female and male time in each group of mice. n.d., the sniffing time could not be determined since mice were completely lethargic. **(F)** Preference index for male mice was calculated as (female time – male time)/ (total sniffing time female + male), as an indicator of the time spent sniffing preferred (female) or non-preferred (male) scents. n.d.: the sniffing time could not be determined since mice were completely lethargic. **(G, H)** Quantification of SARS-CoV-2 RNA in the lungs of IN- (*n* = 7-11, blue dots) and AR- (*n* = 7-10, red dots) infected mice as well as of PBS-treated control mice (*n* = 4, black dots) measured 3 days (**G**) and 6 days (**H**) post infection. RNA values are expressed as copy number per ng of total RNA and the limit of detection is indicated as a dotted line. **(I, J)** Viral titers in the lungs were determined 3 (**I**) and 6 days (**J**) after infection by median tissue culture infectious dose (TCID_50_). PBS-treated control mice: *n* = 2-3, black dots; IN-infected mice: *n* = 4-7, blue dots; AR-infected mice: *n* = 4-10, red dots. **(K)** Representative immunohistochemical (left) and confocal immunofluorescence (right) micrographs of lung sections from PBS-treated control mice (top), IN- (middle) and AR-infected mice (bottom) at 6 days post infection. N-CoV-2 positive cells are depicted in brown (left panels) or in red (right panels). Cell nuclei are depicted in blue (right panels). Scale bars, 30 μm. **(L)** Pulmonary function was assessed by whole-body plethysmography performed 3 and 5 days post IN- (*n* = 6-14, blue dots) and AR-infection (*n* = 7-14, red dots). As control, PBS-treated mice were evaluated (*n* = 6, black dots). Frequency (left) and Rpef (right) parameters are shown. Calculated respiratory values were averaged over a 15 min-data collection period. **(M)** Representative aggregometry curves induced by arachidonic acid (AA) on platelet-rich plasma from PBS-treated control mice (left), IN- (middle) and AR-infected mice (right) 6 days post infection. Platelet aggregation was measured by light transmission aggregometry for 5 min and is expressed as % aggregation. **(N)** Representative immunohistochemical micrographs of lung sections from PBS-treated control mice (left), IN- (middle) and AR-infected mice (right) at 6 days post infection. Fibrin deposition is shown in brown. Scale bars, 30 μm. **(O)** Quantification of the size of fibrin deposits (μm^2^). *n* = 4. Data are expressed as mean ± SEM and are pooled from 2 independent experiments per time point. * p-value < 0.05, ** p-value < 0.01, *** p-value < 0.001; Kruskal-Wallis test (**A, B, E**-**J**); two-way ANOVA followed by Sidak’s multiple comparison test (**L**); Mann-Whitney U-test two-tailed (**D, O**). In **D**, statistical analysis was performed comparing female and male sniffing time within the same experimental group of mice.

We next analyzed viral replication in the upper respiratory tract of K18-hACE2 transgenic mice infected with SARS-CoV-2 via intranasal inoculation or aerosol exposure. We detected the presence of SARS-CoV-2 RNA in the nasal turbinates of both AR- and IN-infected mice at days 3 and 6 p.i. (**Fig. 2A, B**). In order to examine whether viral replication within the upper respiratory tract induced anosmia, we subjected AR- and IN-infected mice to a social scent-discrimination assay (*19*) (**Fig. 2C**). If olfaction is normal (as in PBS-treated controls), male mice exposed to tubes containing male or female bedding preferentially spend time sniffing the female scent (**Fig. 2D-F**). By contrast, both AR- as well as IN-infected mice spent significantly less time sniffing the female scent at day 3 p.i. (**Fig. 2D, E**), indicative of hyposmia or anosmia. Importantly, at day 3 p.i. mobility of both AR- and IN-infected mice was normal, as there were no differences in the amount of time spent sniffing the male tube (**Fig. 2D**). At day 6 p.i., AR-infected mice still showed signs of hyposmia/anosmia, whereas IN-infected mice were completely lethargic preventing further analyses (**Fig. 2D-F**). The data obtained in AR-infected mice are consistent with the hypothesis that hyposmia or anosmia occur because of the infection of olfactory epithelium and in the absence of CNS infection or general malaise (*19*, *28*, *29*).

We next assessed viral replication in the lower respiratory tract of SARS-CoV-2-infected K18-hACE2 transgenic mice. We detected comparable amounts of SARS-CoV-2 RNA and infectious virus from the lungs of mice infected with the two different routes of administration at both day 3 and day 6 p.i. (**Fig. 2G-J**). Immunohistochemical and immunofluorescence staining for the SARS-CoV-2 nucleoprotein confirmed similar levels of viral antigens and similar staining patterns in the lungs of IN- and AR-infected mice (**Fig. 2K**). To gain insight into the impact of infection on lung physiology, pulmonary function was measured at day 3 and 5 p.i. via whole-body plethysmography. Consistent with previously published data (*30*, *31*), we confirmed that, when compared to control mice, IN-infected mice exhibited a significant loss in pulmonary function as indicated by changes in e.g., respiratory frequency, tidal volume, Rpef (a measure of airway obstruction) and PenH (a controversial metric that has been used by some as an indirect measure of airway resistance and by others as a non-specific assessment of breathing patterns (*31*–*34*)) (**Fig. 2L** and **Figure S6A**). Interestingly, most of these respiratory parameters were normal or much less altered in AR-infected mice, suggesting that the observed changes in IN-infected mice were mostly due to CNS infection. One notable exception is Rpef, a calculated index of airway resistance that considers the time needed to reach maximum expiratory flow and the total expiratory time (*31*). Interestingly, Rpef was the only metric that was consistently altered in AR-infected mice (and to the same extent as in IN-infected mice), suggesting that this index might truly reflect lung infection and pathology, rather than CNS involvement.

COVID-19, particularly in its most severe forms, has been associated with thrombotic phenomena that entail increased platelet activation and aggregation, and fibrin deposition in the lungs (*35*–*39*). We therefore set out to assess platelet function by performing light transmission aggregometry of platelet rich plasma (PRP) obtained from infected mice. Whereas IN infection resulted in a significantly impaired platelet aggregation, PRP from AR-infected mice showed a normal or even increased aggregation (**Fig. 2M**). Interestingly, this was associated with increased fibrin deposition and larger platelet aggregates in the lungs of AR-infected mice (**Fig. 2N, O** and **Figure S6B**). Together, the data indicate that aerosol exposure of K18-hACE2 transgenic mice to SARS-CoV-2 results in robust viral replication in the respiratory tract, anosmia, airway obstruction, and platelet aggregation with fibrin deposition in the lung.

Although the vigorous SARS-CoV-2 replication in the lungs of AR-infected K18-hACE2 transgenic mice suggests that these mice *de facto* inhaled at least the same amount of virus as IN-infected mice, it is theoretically possible that a higher viral inoculum would have resulted in fatal neuroinvasion even upon aerosol delivery. To further increase viral replication in the infected hosts, we transiently inhibited type 1 IFN receptor signaling with anti-IFNAR1 blocking antibodies (Abs) prior to AR infection (**Figure S7A**). As expected, higher levels of viral RNA (**Figure S7B**) and infectious virus (**Figure S7C**) were detected in the lungs of mice treated with anti-IFNAR1 Abs compared to control mice. The presence of higher amounts of virus in the lungs of anti-IFNAR1-treated mice was confirmed by immunohistochemical staining for the SARS-CoV-2 nucleoprotein (**Figure S7D**). Despite the increased lung viral load, we failed to detect SARS-CoV-2 RNA, infectious virus, or viral antigens in the brain of anti-IFNAR1-treated AR-infected mice (**Figure S7E-G**).

### Histopathological changes, immune response, and transcriptional signatures in the lungs of infected mice.

**Fig. 3.**
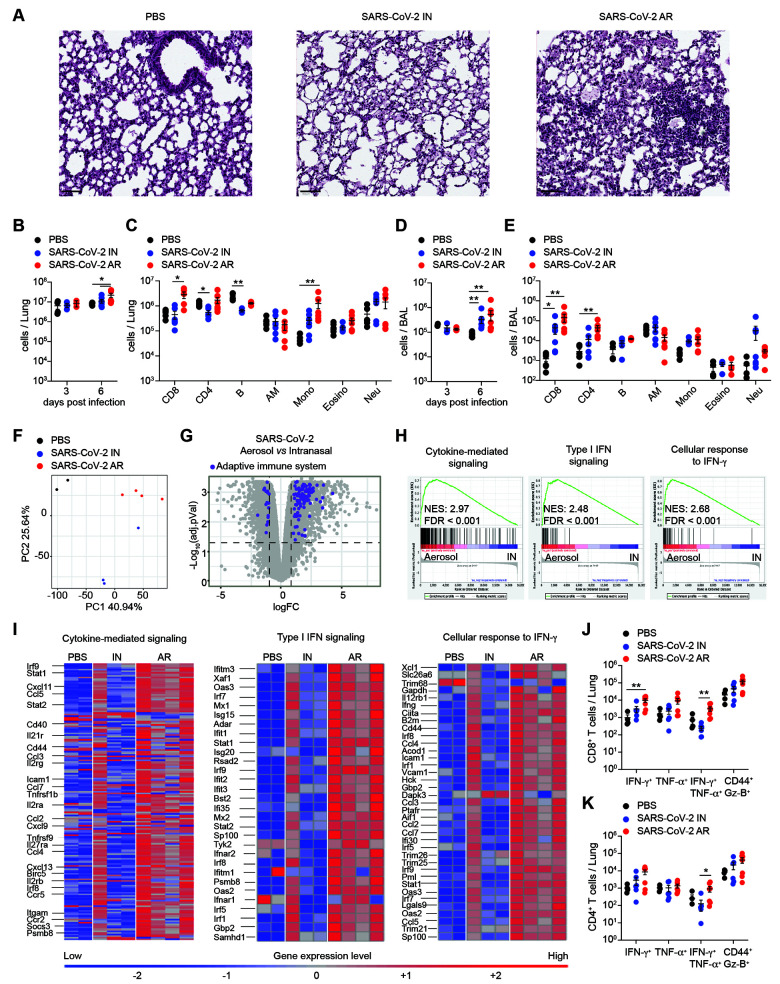
Histopathological changes, immune response, and transcriptional signatures in the lungs of infected mice. **(A)** Representative hematoxylin/eosin (H&E) micrographs of lung sections from PBS-treated control mice (left), IN- (middle) and AR-infected mice (right) 6 days post infection. Scale bars, 50 μm. **(B-E)** Absolute number of total cells (**B, D**) and of single cell populations (**C, E**) recovered from lung homogenates (**B, C**) and bronchoalveolar lavage (BAL) (**D, E**) of PBS-treated control mice (*n* = 4-6, black dots), IN- (*n* = 3-7, blue dots) and AR-infected mice (*n* = 3-7, red dots) analyzed 6 days post infection. CD8^+^ T cells (Live, CD45^+^, CD8^+^); CD4^+^ T cells (Live, CD45^+^, CD4^+^); B cells (Live, CD45^+^, CD8^-^, CD4^-^, B220^+^, CD19^+^); AM, alveolar macrophages (Live, CD45^+^, CD8^-^, CD4^-^, Ly6g^-^, CD11b^-^, F4/80^+^, SiglecF^hi^); Mono, monocytes (Live, CD45^+^, CD8^-^, CD4^-^, Ly6g^-^, SiglecF^-^, CD11b^+^, Ly-6c^+^); Eosino, eosinophils (Live, CD45^+^, CD8^-^, CD4^-^, Ly6g^-^, CD11b^+^, SiglecF^int^); Neu, neutrophils (Live, CD45^+^, CD8^-^, CD4^-^, CD11b^+^, Ly6g^+^). **(F)** Principal Component Analysis (PCA) of RNA-seq expression values from the lungs of PBS-treated, control (*n* = 2, black), IN- (*n* = 3, blue) and AR-infected (*n* = 4, red) mice. Percentages indicate the variance explained by each PC. **(G)** Volcano plot of RNA-Seq results. The X-axis represents the Log2 Fold-Change of Differentially Expressed Genes (DEG) comparing AR- to IN-infection, the Y-axis the -Log10(FDR). Genes significantly up-regulated in AR- relative to IN- (|logFC| > 1 and adjusted P value < 0.05, horizontal and vertical dashed line) belonging to the “Adaptive immune system” pathway from BioPlanet 2019 (*55*) are highlighted in violet. **(H)** GSEA of three gene sets described in (*18*), comparing the transcriptome of IN- and AR-infected mice, presented as the running enrichment score for the gene set as the analysis 'walks down' the ranked list of genes (reflective of the degree to which the gene set is over-represented at the top or bottom of the ranked list of genes) (top), the position of the gene-set members (black vertical lines) in the ranked list of genes (middle) and the value of the ranking metric (bottom). **(I)** Heatmaps of genes (one per row) belonging to the three signatures as in (**H**), expressed in logarithmic normalized read counts. Each column represents an individual sample. **(J, K)** Absolute number of CD8^+^ (**J**) and CD4^+^ (**K**) T cells producing IFN-γ, TNF-α or both and expressing CD44 and Granzyme-B (Gz-B) in the lungs of PBS-treated control mice (*n* = 4, black dots), IN- (*n* = 5, blue dots) and AR-infected mice (*n* = 5, red dots) 6 days after infection. Data are expressed as mean ± SEM. Data in (B-E, J, K) are pooled from 2 independent experiments. * p-value < 0.05, ** p-value < 0.01; Kruskal-Wallis test (**B-E, J, K**).

We next sought to better characterize the histopathological changes and the immune response in the lungs of SARS-CoV-2 infected mice. Hematoxylin and eosin staining revealed an inflammatory process that peaked at day 6 p.i. and appeared more severe in AR- than in IN-infected mice (**Fig. 3A** and **Figure S8A**). Lung sections revealed interstitial edema, consolidation, alveolar wall thickening and immune infiltration of both polymorphonuclear and mononuclear cells in the alveolar as well as the interstitial space (**Fig. 3A** and **Figure S8A**). Consistent with the histology, the absolute number of cells recovered 6 days after infection from the lungs and bronchoalveolar lavage (BAL) was significantly higher in AR-infected than in IN-infected mice (**Fig. 3B-E**). Specifically, the differences in immune cell recruitment could be attributed to an increase in CD4^+^ and CD8^+^ T cells as well as in monocytes (**Fig. 3B-E**). A higher number of TCR-β^+^ T cells in the lung of AR-infected mice was confirmed by confocal immunofluorescence histology (**Figure S8B**).

Next, we sought to analyze the transcriptome in the lungs of infected K18-hACE2 mice by performing bulk RNA sequencing (RNA-seq) of lung homogenates 6 days after SARS-CoV-2 infection. Principal component analysis revealed distinct transcriptional signatures between AR-infected, IN-infected, and uninfected mice (**Fig. 3F**). Genes up-regulated upon AR infection were associated to adaptive immune responses, and to immune system signaling by interferons and other cytokines (**Fig. 3G**, **Figure S9** and **Data file S1**). In particular, the transcription of genes related to cytokine-mediated signaling, type I IFN signaling and cellular response to IFN-γ (*18*) was increased in the lungs of AR-infected mice with respect to IN-infected ones (**Fig. 3H, I** and **Data file S1**). Interestingly, many human orthologs of the genes up-regulated in the lungs of AR-infected mice were found to be also induced in COVID-19 patients, including genes related to leukocyte trafficking (e.g., *Ccl11*, *Ccl8*, *Ccl2*, *Cxcl9* and *Cxcl10*), antiviral response induced by type I IFN (*Ifitm3*, *Ifit2*, *Oas1a*, *Oas3*, *Stat1*, *Irf1*, *Mx1*, *Mx2*, *Isg15*) and TNF (*Tnfsf10*) (*40*–*42*)(**Fig. 3I****, Figure S9** and **Data file S1**). Of note, *Ccl8* and *Ccl2* as well as *Cxcl9* and *Cxcl10*, chemoattractants for monocytes and T cells, respectively, were significantly up-regulated in the lungs of AR-infected mice and in COVID-19 patients (*40*–*42*), in line with the increased recruitment of these cells (**Fig. 3I**, **Figure S9C** and **Data file S1**). The enrichment of the gene signature related to the cellular response to IFN-γ in AR-infected mice prompted us to assess SARS-CoV-2-specific T cell responses. Antigen-specific CD8^+^ and CD4^+^ T cells recovered from lung homogenates were assessed for IFN-γ, TNF-α and Granzyme-B (Gz-B) expression upon in vitro stimulation with the H2-D^b^-restricted S538-546 and I-A^b^-restricted ORF3a 266-280 immunodominant peptides (*43*). In line with the RNA-seq data, we found that the absolute number of IFN-γ^+^ TNF-α^+^ virus-specific CD8^+^ and CD4^+^ T cells were significantly higher in the lungs of AR-infected mice compared to IN-infected mice (**Fig. 3J, K**). Taken together, our data show that, when compared to intranasal inoculation, aerosol infection resulted in a more pronounced lung pathology including increased immune infiltration, and in a transcriptional signature comparable to that observed in SARS-CoV-2-infected patients.

## DISCUSSION

We have generated and characterized an alternative COVID-19 platform, based on controlled aerosol exposure of K18-hACE2 transgenic mice to SARS-CoV-2. Mice infected via aerosol develop robust respiratory infection, anosmia, and signs of airway obstruction but, in contrast to mice infected intranasally, do not experience fatal neuroinvasion. Moreover, when compared to intranasal inoculation, aerosol exposure results in a more severe lung pathology, inflammation, and fibrin deposition.

The observation that mice infected via aerosol exposure develop anosmia in the absence of neuroinvasion is of interest in light of the notion that olfactory dysfunction is a strong and consistent symptom associated with a positive COVID-19 test in humans (*44*). The pathophysiology of anosmia triggered by SARS-CoV-2 remains unclear. However, our results, as well as previously published studies (*19*, *28*, *29*, *45*–*47*), support the hypothesis that anosmia stems from infection of sustentacular cells and/or Bowman’s glands rather than of olfactory sensory neurons. Indeed, sustentacular cells and Bowman’s glands in both K18-hACE2 transgenic mice and humans, express high levels of the SARS-CoV-2 receptor ACE2 as well as the internalization enhancer TMPRSS2 (*47*). Infected sustentacular cells and/or Bowman’s glands may in turn produce pro-inflammatory cytokines affecting olfactory sensory neurons (*29*, *48*). Alternatively, damaged sustentacular cells and/or Bowman’s glands may lead to an overall disorganization of the olfactory epithelium, ultimately leading to defective signal transduction to the olfactory bulb (*49*–*51*). Elucidation of the molecular mechanisms underlying anosmia during COVID-19 will require further studies.

The difference in immune infiltration in the lungs between intranasal and aerosol infection (despite similar viral loads) remains poorly understood. A possible explanation lies in central nervous system injury causing immune deficiency (*52*). Similarly, the observed failure of platelet aggregation might be influenced by central nervous system damage (*53*). Previous studies have looked at how the route of inoculation affects the immune response and disease outcome upon viral infection. For instance, one study directly compared intranasal instillation with aerosol inoculation of mice with influenza virus and concluded that aerosol delivery resulted in a more robust infection, pulmonary cell infiltration and inflammation, and morbidity (*54*).

We believe that this model may allow studies on viral transmission (e.g., by analyzing the effect of aerosol particle size, humidity, and temperature on infectivity), on disease pathogenesis (including, potentially, thrombotic events and long-term consequences of infection) and on therapeutic interventions.

## MATERIALS AND METHODS

Please see Supplementary Methods for additional details.

### Study design

The aim of this study was to develop mouse models of COVID-19 pathogenesis and treatment. We used SARS-CoV-2/human/ITA/Milan-UNIMI-1/2020 as virus, K18-hACE2 transgenic mice as model organism, and a nose-only inhalation tower system to expose mice to aerosolized virus under controlled conditions. Mouse experiments were planned in accordance with the principles of the 3Rs (replacement, reduction, and refinement). Body weight, morbidity and mortality were recorded. Olfactory and respiratory functions were measured by social scent discrimination assay and by whole-body plethysmography, respectively. Viral content in different organs was measured by tissue culture infectious dose 50, by qPCR, by immunohistochemistry and by immunofluorescence. Immune infiltrate and pathological changes were examined in different organs by flow cytometry and by histology/immunohistochemistry, respectively. Platelet function was measured via light aggregometry and fibrin deposition by immunohistochemistry. Gene expression was analyzed by qPCR and RNA-sequencing. Sample size and replicates for each experiment are indicated in figure legends. During analysis, no individual data points were excluded under any circumstances other than technical failure to process the sample. Animals were randomized to the experimental groups.

### Mice

B6.Cg-Tg(K18-ACE2)^2Prlmn/^J mice (referred to in the text as K18-hACE2) were purchased from The Jackson Laboratory. Mice were housed under specific pathogen-free conditions and heterozygous mice of both sexes were used at 8-10 weeks of age. All experimental animal procedures were approved by the Institutional Animal Committee of the San Raffaele Scientific Institute and all infectious work was performed in designed BSL-3 workspaces.

### Virus

The SARS-CoV-2/human/ITA/Milan-UNIMI-1/2020 (GenBank: MT748758.1) isolation was carried out in BSL-3 workspace and performed in Vero E6 cells, which were cultured at 37°C, 5% CO2 in complete medium (DMEM supplemented with 10% FBS, MEM non-essential amino acids, 100 U/ml penicillin, 100 U/ml streptomycin, 2mM L-glutamine). Virus stocks were titrated using Endpoint Dilutions Assay (EDA, TCID50/ml). Vero E6 cells were seeded into 96 wells plates and infected at 95% of confluency with base 10 dilutions of virus stock. After 1h of adsorption at 37°C, the cell-free virus was removed, cells were washed with PBS 1X, and complete medium was added to cells. After 48h, plates were evaluated for the presence of a cytopathic effect (CPE). TCID50/ml of viral stocks were then determined by applying the Reed–Muench formula.

### Nose-only inhalation tower system

The DSI nose-only inhalation tower system (DSI Buxco respiratory solutions, DSI) is composed of 7 open ports where mice are exposed to aerosolized virus. Mice were placed in the DSI Allay restraint. The Allay collar is positioned between the base of the mouse skull and its shoulders, thus avoiding thorax compression, and maintaining normal breathing patterns. Liquid virus was aerosolized by an Aeroneb (Aerogen) vibrating mesh nebulizer that generates particles of ~4 μm size which were uniformly delivered to all the tower ports. One port of the tower was occupied by a temperature and humidity probe for real-time monitoring of the tower conditions. The inhalation tower controller software was used to define the flow and pressure of the inhalation tower as well as the temperature, humidity, and the ratio O_2_/CO_2_ inside the tower. During virus exposure mice were monitored through plethysmography for frequency, tidal volume, minute volume and accumulated volume. The whole system was placed inside a class II biological safety hood that is located in a BSL-3 facility.

### Mouse infection through aerosol exposure (AR) or intranasal administration (IN)

Unanesthetized K18-hACE2 mice were placed in a nose-only Allay restrainer on the inhalation chamber (DSI Buxco respiratory solutions, DSI). To reach a target accumulated inhaled aerosol (also known as delivered dose) of 1 × 10^5^ TCID50, mice were exposed to aerosolized SARS-CoV-2 for 20-30 min (depending on the total volume of diluted virus and on the number of mice simultaneously exposed). Primary inflows and pressure were controlled and set to 0,5 L/minute/port and -0,5 cmH_2_O, respectively. As control, K18-hACE2 mice received the same volume of aerosolized PBS (125 μL per mouse). Intranasal administration of 1 × 10^5^ TCID50 of SARS-COV-2 per mouse in a total volume of 25 μL PBS was performed under 2% isoflurane (#IsoVet250) anesthesia. Infected mice were monitored daily to record body weight, clinical and respiratory parameters. The clinical score was based on a cumulative 0-3 scale evaluating fur, posture, activity level, eyes and breathing (see Table S1).

### Social scent-discrimination test

The social scent-discrimination test was used to assess hyposmia/anosmia in IN-infected or AR-infected male mice compared to PBS-treated control male mice, as previously described (*19*). Briefly, two 2 ml-Eppendorf tubes containing beddings from the cage of grouped females and from the experimental male were placed at two opposing corners of a clean cage. Experimental male mice were scored for 5 min for the time spent in sniffing the tubes, considering only the time when the nose was inside one of the two tubes. Control male mice preferentially explored the tube containing the female bedding. Preference index was calculated as: (time spent to sniff female tube - time spent to sniff male tube) / (time spent to sniff female tube + time spent to sniff male tube).

### Whole-body plethysmography

Whole-body plethysmography (WBP) was performed using WBP chamber (DSI Buxco respiratory solutions, DSI). Mice were allowed to acclimate inside the chamber for 10 min before recording respiratory parameters for 15 min using the FinePointe software.

### Statistical analyses and software

Detailed information concerning the statistical methods used is provided in the figure legends. Flow and imaging data were collected using FlowJo Version 10.5.3 (Treestar) and Imaris (Bitplane), respectively. Statistical analyses were performed with GraphPad Prism software version 8 (GraphPad). Immunohistochemical imaging quantifications were performed with QuPath (Quantitative Pathology & Bioimage 5 Analysis) software. *n* represents individual mice analyzed per experiment. Experiments were performed independently at least twice to control for experimental variation. Error bars indicate the standard error of the mean (SEM). We used Mann-Whitney U-tests to compare two groups with non-normally distributed continuous variables and Kruskal-Wallis non-parametric test to compare three or more unpaired groups. We used two-way ANOVA followed by Sidak’s multiple comparisons tests to analyze experiments with multiple groups and two independent variables. Kaplan-Meier curves were compared with the Log-rank (Mantel-Cox) test. Significance is indicated as follows: *p < 0.05; **p < 0.01; ***p < 0.001. Comparisons are not statistically significant unless indicated.

## Supplementary Materials

### This PDF file includes:

Supplementary Methods Figs. S1 to S9 Tables S1 and S2 References (*56*–*65*)

### Other Supplementary Material for this manuscript includes the following:

Data files S1 and S2

MDAR Reproducibility Checklist
