# Supplementary material for: Administration of aerosolized SARS-CoV-2 to K18-hACE2 mice uncouples respiratory infection from fatal neuroinvasion
Source: Sci Immunol. 2021 Nov 23:eabl9929. doi: 10.1126/sciimmunol.abl9929 (PMC9835999; doi:10.1126/sciimmunol.abl9929)
Supplement: Supplementary file 1 — Supplementary Methods Figs. S1 to S9 Tables S1 and S2 References (56–65) [file sciimmunol.abl9929_sm.pdf]

Science Immunology
AAAS

Supplementary Materials for

Administration of aerosolized SARS-CoV-2 to K18-hACE2 mice uncouples respiratory infection from fatal neuroinvasion

Valeria Fumagalli *et al.*

Corresponding author: Matteo Iannacone, iannacone.matteo@hsr.it; Luca G. Guidotti, guidotti.luca@hsr.it

DOI: 10.1126/sciimmunol.abl9929

The PDF file includes:

Supplementary Methods
Figs. S1 to S9
Tables S1 and S2
References (*56–65*)

Other Supplementary Material for this manuscript includes the following:

Data files S1 and S2
MDAR Reproducibility Checklist

Supplementary Methods

In vivo treatment

In selected experiments, K18-hACE2 mice were injected intraperitoneally with 2 mg per mouse of α-IFNAR1 blocking antibody (BioXcell, #BE0241, clone MAR1-5A3) 1 day before infection.

Platelet aggregation

Blood was collected from the retro-orbital sinus into 1:10 volume of citrate phosphate dextrose (CPD; Sigma-Aldrich, #C7165) and platelet-rich plasma (PRP) was prepared as described (*56*). The PRP platelet count was adjusted to the lowest value of the day. Homologous platelet-poor plasma (PPP) was isolated by spinning the remaining peripheral blood from PRP at 3500 rpm for 10 minutes. Aggregation in stirred PRP at 37°C was induced by adding 125 μM of Arachidonic Acid (Mascia Brunelli, #311501WB) and monitored by recording changes in light transmittance through the PRP suspension using a Chrono-log model 490 aggregometer (Chrono-log Corporation, Havertown, PA).

RNA extraction and qPCR

Tissues homogenates were prepared by homogenizing perfused lung, brain, olfactory bulb, and nasal turbinates using gentleMACS dissociator (Miltenyi BioTec, #130-096-427) with program RNA_02 in M tubes (#130-096-335) in 1 ml of Trizol (Invitrogen, #15596018). The homogenates were centrifuged at 2000 g for 1 min at 4°C and the supernatant was collected. RNA extraction was performed by combining

phenol/guanidine-based lysis with silica membrane-based purification. Briefly, 100 μl of Chloroform were added to 500 μl of homogenized sample and total RNA was extracted using ReliaPrep™ RNA Tissue Miniprep column (Promega, Cat #Z6111). Total RNA was isolated according to the manufacturer's instructions. qPCR was performed using TaqMan Fast virus 1 Step PCR Master Mix (Lifetechnologies #4444434), standard curve was drawn with 2019_nCOV_N Positive control (IDT#10006625), primer used are: 2019-nCoV_N1- Forward Primer (5'-GAC CCC AAA ATC AGC GAA AT-3'), 2019-nCoV_N1- Reverse Primer (5'-TCT GGT TAC TGC CAG TTG AAT CTG-3') 2019-nCoV_N1-Probe (5'-FAM-ACC CCG CAT TAC GTT TGG ACC-BHQ1-3') (Centers for Disease Control and Prevention (CDC) Atlanta, GA 30333). All experiments were performed in duplicate.

RNA-seq library preparation

Total RNA was obtained from homogenized lung tissues, as described above, for bulk RNA sequencing. Sequencing libraries were generated using Smart-seq2 method, as described (*57*, *58*). In brief, 4 ng of RNA were retrotranscribed and cDNA was amplified using 13 cycles and purified with AMPure XP beads (Beckman Coulter). Concentration was determined using Qubit 3.0 (Life Technologies) and the size distribution was assessed using Agilent 4200 TapeStation system. The following tagmentation reaction was performed starting from 0.5 ng of cDNA for 30 min at 55°C and the enrichment PCR was carried out using 12 cycles. Libraries were then purified with AMPure XP beads, quantified using Qubit 3.0 and single-end sequenced (75 bp) on an Illumina NextSeq 500.

RNA-seq data processing and analysis

Raw reads were aligned to mouse genome build GRCm38 using STAR aligner (*59*). Gene counts were generated using featureCounts (part of the Subread package (*60*)), based on GENCODE gene annotation version M22. In order to discard genes highly expressed by one sample only, a filter of cpm ≥ 2 in at least two samples was added. Read counts were normalized with the Trimmed Mean of M-values (TMM) method (*61*) using calcNormFactors function and then Voom (*62*) was applied. Differentially Expressed Genes (DEGs) between AR- and IN-infected mice were identified by generating a linear model using LIMMA R package (*63*) (**Figure S9** and **Data file S1**).

Gene Set Enrichment Analysis and signatures visualization

Gene Set Enrichment Analysis (GSEA) was performed using the GseaPreranked Java tool (*64*) with pre-ranked Log2 fold changes between aerosol and intranasal samples in expressed genes. Three signatures described in (*18*) were analyzed.

Histochemistry

Mice were euthanized and perfused transcardially with PBS. One left lobe of the lung and a sagittal section of a hemisphere of the brain were fixed in zinc formalin and transferred into 70% ethanol 24h later. Tissues were then processed, embedded in paraffin, and automatically stained for SARS-CoV-2 (2019-nCoV) Nucleocapsid (SINO BIO, #40143-R019) or for fibrin (DAKO, #A0080) through LEICA BOND RX 1h room-temperature (RT) and developed with Bond Polymer Refine Detection (Leica, DS9800). Note that the antibody against N-SARS-CoV-2 gives unspecific signal on terminal bronchioles even in PBS-treated control mice. For

hematoxylin and eosin (H&E) staining, tissues were stained as previously described (*57*, *65*). Bright-field images were acquired with an Aperio Scanscope System CS2 microscope and the ImageScope program (Leica Biosystem) following the manufacturer's instructions. N-SARS-CoV-2 percentage of positive area (**Figure S7 D, G**) was determined by the QuPath (Quantitative Pathology & Bioimage 5 Analysis) software. Size of fibrin deposits (**Figure 2O**) was determined by automatically building masks on fibrin-positive areas and calculating the covered surface that was expressed in μm^2 with the QuPath software.

<u>Confocal Immunofluorescence Histology</u>

Mice were euthanized and perfused transcardially with PBS. One left lobe of the lung was collected and fixed in 4% paraformaldehyde for 16h, then dehydrated in 30% sucrose prior to embedding in OCT freezing media (Killik Bio-Optica #05-9801). 20 μm sections were cut on a CM1520 cryostat (Leica) and adhered to Superfrost Plus slides (Thermo Scientific). Sections were permeabilized and blocked in PBS containing 0.3% Triton X-100 (Sigma-Aldrich) and 0,5% BSA followed by staining in PBS containing 0.1% Triton X-100 and 0,2% BSA. Slides were stained for SARS-CoV-2 nucleocapsid (GeneTex, polyclonal, #GTX135357), CD41 (Biolegend, Clone MWReg30, #133908) or TCRβ (Biolegend, Clone H57-597, #109218) for 1h at room temperature. Then, slides were stained with Alexa Fluor 568-conjugated goat anti-rabbit IgG (Life Technologies, #A-11011) for 2h at room temperature (for SARS-CoV-2 nucleocapsid staining). A hemisphere of the brain was fixed in 4% paraformaldehyde for 48h, then dehydrated in 30% sucrose for 24h. Prior to embedding in OCT freezing media, brain was soaked in a solution 1:1 of 30% sucrose and OCT for 1h in agitation. Brain was cut in 50 μm-thick sagittal sections

and free-floating sections were rinsed in PBS/Azide 1%. Quenching was performed by shaking (300rpm) the sections for 10 min at room temperature in a PBS solution containing 1:100 methanol and 1:30 H_2O_2. The sections were then incubated 20 min with 0.3% Triton X-100 and 0.5% BSA for 1 h. Immunofluorescence staining was performed in PBS containing 0.1% Triton X-100 and 0.2% BSA over night (O/N) at 4°C and then stained with secondary antibody for 2h at room temperature. The following primary Abs were used for staining: anti-SARS-CoV-2 nucleocapsid (GeneTex, #GTX135357), anti-NeuN (Merck Millipore, #MAB377), anti-GFAP (Abcam, #ab4674), anti-Iba1 (Synaptic system, #234006), anti-CD68 (Abcam #ab53444), and anti-iNOS (Abcam #ab3523). The following secondary Abs were used for staining: Alexa Fluor 647-conjugated goat anti-chicken IgG (Thermo Fisher, #A-21), Alexa Fluor 568-conjugated goat anti-rabbit IgG (Thermo Fisher, #A-11011), Alexa Fluor 488-conjugated rat anti-mouse IgG (BD Bioscience, #553443). Lung and brain sections were washed twice for 5 min and stained with DAPI (Life technologies, #D1360) for 5 min at RT, then washed again and mounted for imaging with FluorSaveTM Reagent (Merck Millipore, #345789). Images were acquired on an SP5 or SP8 confocal microscope with 20x objective (Leica Microsystem). To minimize fluorophore spectral spillover, the Leica sequential laser excitation and detection modality was used.

Supplementary Figures

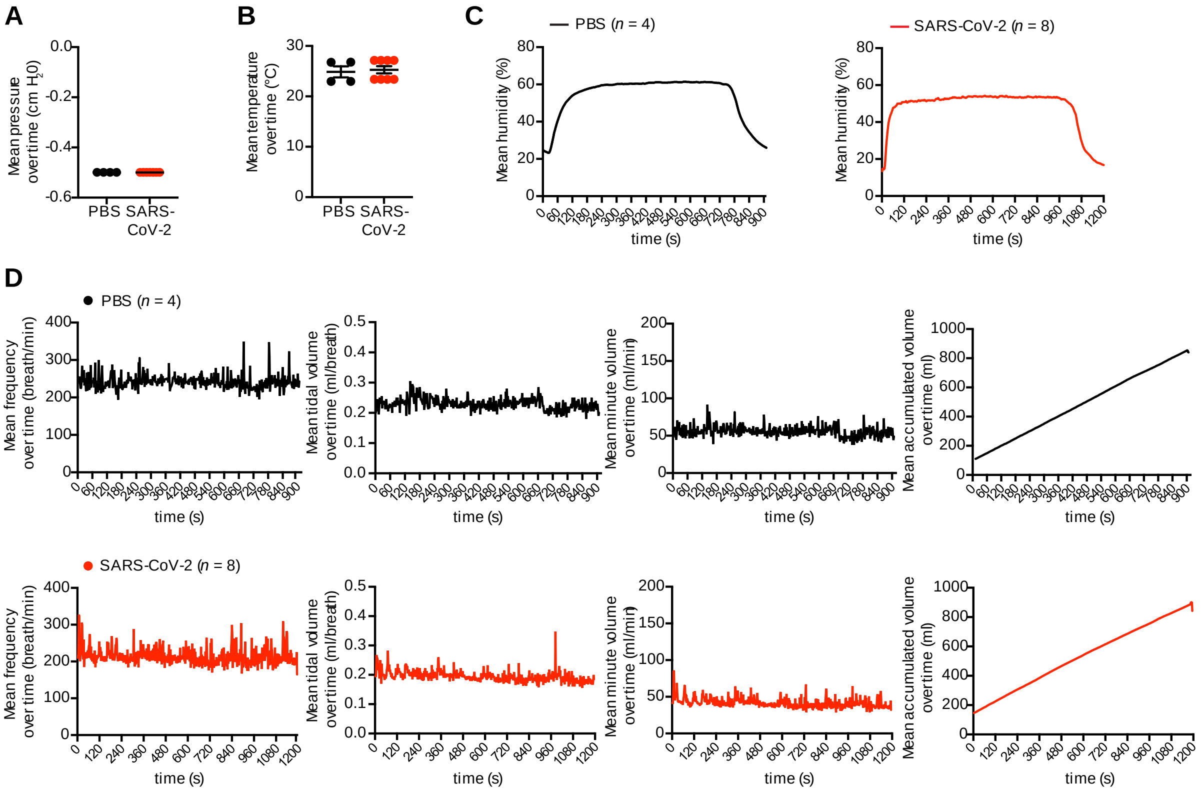

Figure S1. ***Recorded parameters in the nose-only inhalation tower system.*** **(A)** Mean of the pressure (cm H2O) measured within the outer core of the nose-only inhalation tower during the time of PBS-exposure (*n* = 4, black dots) or SARS-CoV-2-exposure (*n* = 8, red dots) of K18-hACE2 mice. **(B)** Mean temperature (°C) over time at a chamber port exposed to PBS (*n* = 4, black dots) or SARS-CoV-2 (*n* = 8, red dots). **(C)** Mean humidity measured over time at a chamber port exposed to PBS (left panel, *n* = 4, black line) or SARS-CoV-2 (right panel, *n* = 8, red line). **(D)** Respiratory parameters analyzed during exposure through plethysmography associated to the Allay restrainer. Mean of breathing frequency, tidal volume, minute volume and accumulated inhaled volume measured during mouse exposure to PBS (upper panels, *n* = 4, black lines) or SARS-CoV-2 (lower panels, *n* = 8, red lines). Data are expressed as mean ± SEM (**A, B**) and as mean (**C-D**) and are representative of at least 2 independent experiments.

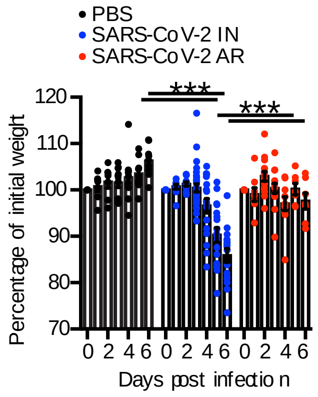

Figure S2. ***Body weight change upon intranasal (IN) or aerosol (AR) infection of K18-hACE2 transgenic mice with SARS-CoV-2.*** Mouse body weight related to the experiment described in Figure 1C, representing each mouse as an individual point.

Data are expressed as mean ± SEM. *** p-value < 0.001; Two-way ANOVA followed by Sidak's multiple comparison test.

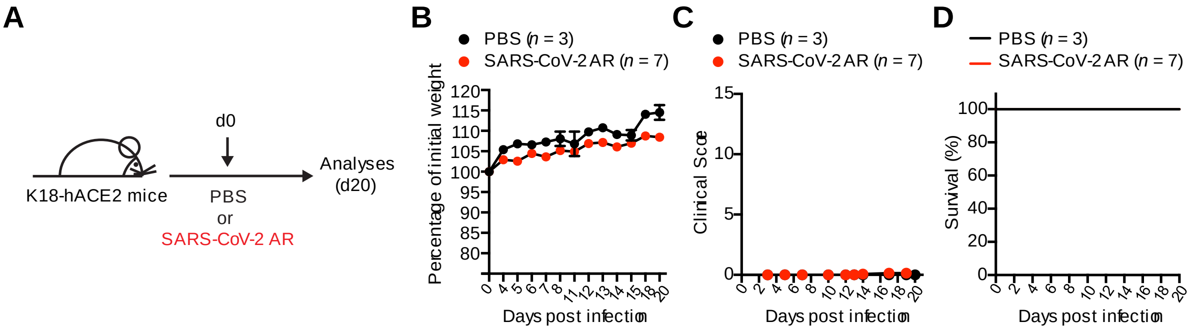

Figure S3. ***Body weight, clinical score and survival of K18-hACE2 mice 20 days after aerosol (AR) exposure to SARS-CoV-2.*** **(A)** Schematic representation of the experimental setup. K18-hACE2 mice were infected with a target dose of 1 x 10^5 $TCID_{50}$ of SARS-CoV-2 through aerosol (AR) exposure. Analyses were performed 20 days post infection. **(B)** Mouse body weight was monitored daily for up to 20 days and is expressed as the percentage of weight relative to the initial weight on day 0. Control mice treated with PBS: black dots (*n* = 3); AR-infected mice: red dots (*n* = 7). **(C)** Clinical score was assessed evaluating the piloerection (0-3), posture (0-3), activity level (0-3), eye closure (0-3) and breathing (0-3). **(D)** Survival curve of indicated mice is shown.

Data are represented as mean ± SEM and are representative of 2 independent experiments.

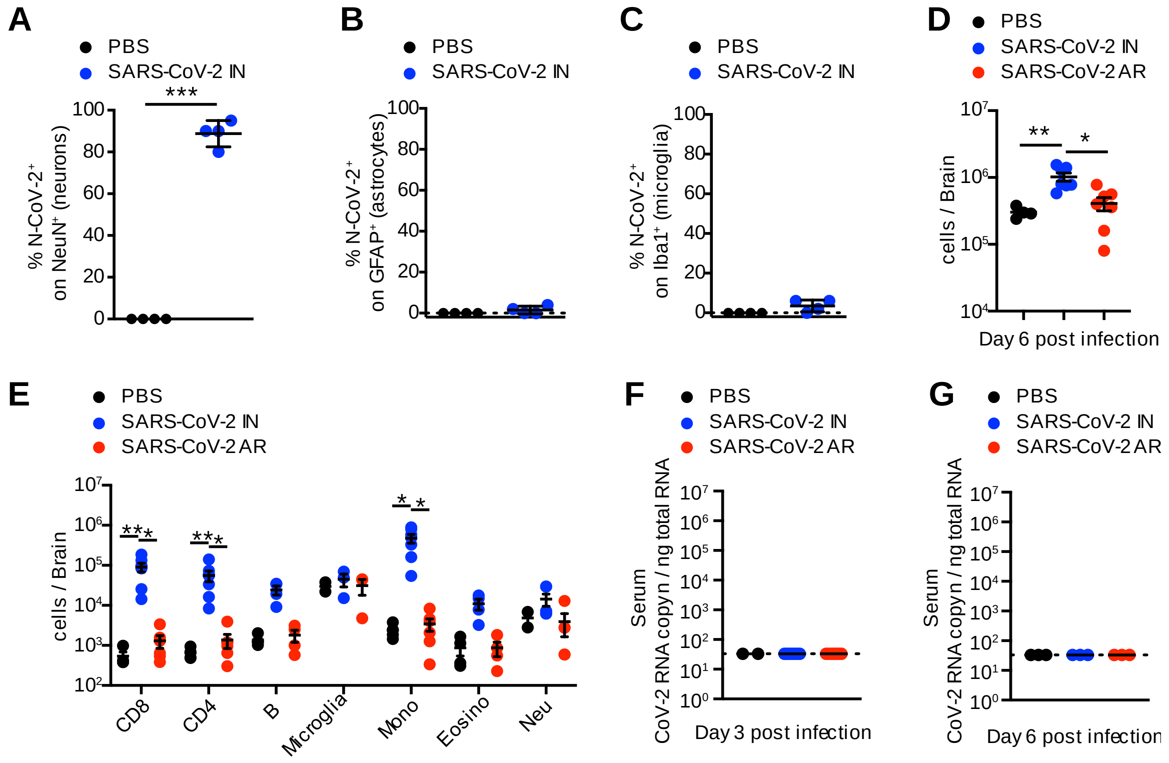

Figure S4. *SARS-CoV-2 neuroinvasion occurs upon intranasal infection, but not upon aerosol exposure.* (A) Quantification (corresponding to Figure 1L) of neurons infected with SARS-CoV-2 as percentage of N-CoV-2$^+$ cells on total NeuN$^+$ cells in the cerebral cortex of PBS-treated control mice (*n* = 4, black dots) and IN-infected mice (*n* = 4, blue dots) 6 days post infection. **(B)** Quantification (corresponding to Figure 1N) of astrocytes infected with SARS-CoV-2 as percentage of N-CoV-2$^+$ cells on total GFAP$^+$ cells in the cerebral cortex of PBS-treated control mice (*n* = 4, black dots) and IN-infected mice (*n* = 4, blue dots) 6 days post infection. **(C)** Quantification (corresponding to Figure 1O) of infected microglia as percentage of N-CoV-2$^+$ cells on the total Iba1$^+$ cells in the cerebral cortex of PBS-treated control mice (*n* = 4, black dots) and IN-infected mice (*n* = 4, blue dots) 6 days post infection. **(D, E)** Absolute number of total cells (**D**) and single cell population (**E**) recovered from brain homogenates of PBS-treated control mice (*n* = 4, black dots), IN- (*n* = 3-7, blue dots) and AR-infected mice (*n* = 3-7, red dots) analyzed 6 days post infection. CD8$^+$ T cells (Live, CD45^{hi}, CD8$^+$); CD4$^+$ T cells (Live, CD45^{hi}, CD4$^+$); B cells (Live, CD45^{hi}, CD8$^-$, CD4$^-$, B220$^+$, CD19$^+$); microglia (Live, CD45^{int}, CD64$^+$, F4/80$^+$); Mono, monocytes (Live, CD45^{hi}, CD8$^-$, CD4$^-$, Ly6g$^-$, SiglecF$^-$, CD11b$^+$, Ly-6c$^+$); Eosino, eosinophils (Live, CD45^{hi}, CD8$^-$, CD4$^-$, Ly6g$^-$, CD11b$^+$, SiglecF^{int}); Neu, neutrophils (Live, CD45^{hi}, CD8$^-$, CD4$^-$, CD11b$^+$, Ly6g$^+$). **(F, G)** Quantification of SARS-CoV-2

RNA in the serum of IN- (*n* = 4, blue dots) and AR- (*n* = 4, red dots) infected mice as well as of PBS-treated control mice (*n* = 3, black dots) measured 3 days (**F**) and 6 days (**G**) post infection. RNA values are expressed as copy number per ng of total RNA and the limit of detection is indicated as a dotted line.
Data are expressed as mean ± SEM. Data in (D-E) are pooled from 2 independent experiments per time point. * p-value < 0.05, ** p-value < 0.01, *** p-value < 0.001; Mann-Whitney U-test two-tailed (**A-C**); Kruskal-Wallis test (**D-G**).

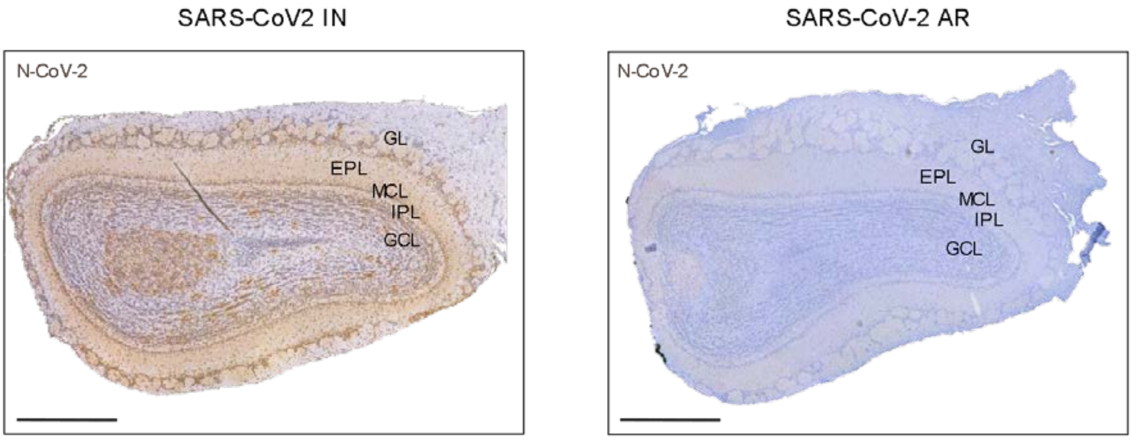

Figure S5. ***SARS-CoV-2 RNA was detected in the olfactory bulb of IN-infected, but not AR-infected K18-hACE2 mice*.**

Representative immunohistochemical micrographs of coronal sections of the olfactory bulbs from intranasal (IN)- (left) and aerosol (AR)-infected mice (right) at 6 days post infection. N-CoV-2 positive cells are depicted in brown. GL, glomerular layer; EPL, external plexiform layer; MCL, mitral cell layer; IPL, internal plexiform layer; GL, granular cell layer. Scale bars, 500 µm.

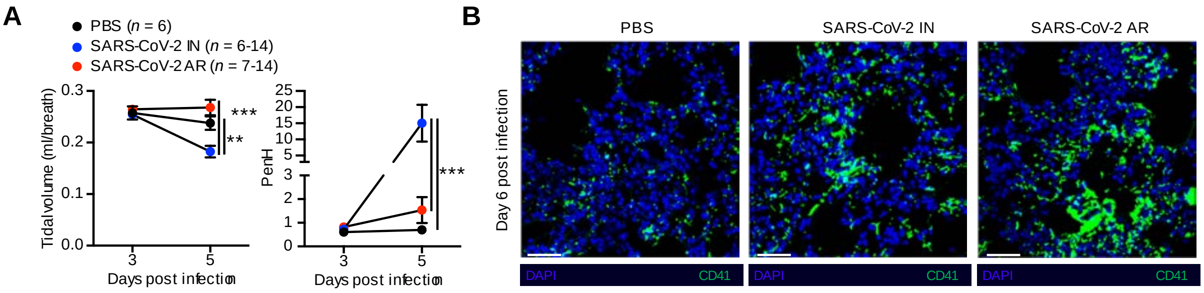

Figure S6. *Breathing parameters and platelet aggregates in the lungs of SARS-CoV-2 infected mice.* (A) Pulmonary function was assessed by whole-body plethysmography performed 3 and 5 days post IN- (*n* = 6-14, blue dots) and AR-infection (*n* = 7-14, red dots). As control, PBS-treated mice were evaluated (*n* = 6, black dots). Tidal volume (left) and PenH (right) parameters are shown. Calculated respiratory values were averaged over a 15 minute data collection period. **(B)** Representative confocal immunofluorescence micrographs of lung sections from PBS-treated control mice (left), IN- (middle) and AR-infected mice (right) 6 days post infection. $CD41^{+}$ platelets are depicted in green; cell nuclei are depicted in blue. Scale bars, 30 μm.

Data are expressed as mean ± SEM and are pooled from 2 independent experiments per time point. ** p-value < 0.01, *** p-value < 0.001; two-way ANOVA followed by Sidak's multiple comparison test.

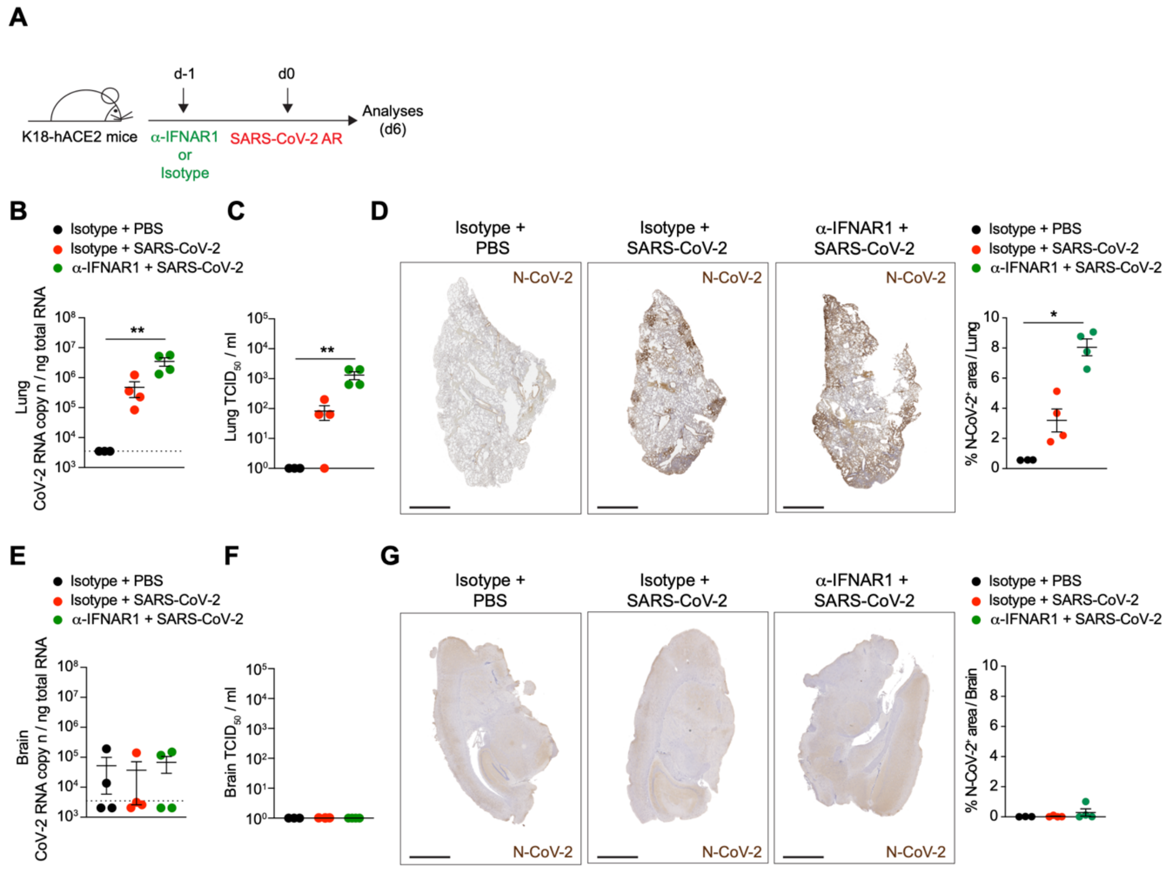

Figure S7. ***Aerosol exposure of K18-hACE2 transgenic mice to SARS-CoV-2 does not lead to fatal neuroinvasion even upon type I IFN receptor blockade.*** **(A)** Schematic representation of the experimental setup. K18h-ACE2 mice were treated with anti-IFNAR1 blocking antibody (or isotype control) 1 day before infection with a target dose of 1 x 10^5 $TCID_{50}$ of SARS-CoV-2 via aerosol exposure. Lungs and brains were collected and analyzed 6 days post infection. **(B, E)** Quantification of SARS-CoV-2 RNA in the lungs (**B**) and brains (**E** of isotype control- (*n* = 4, red dots) and α–IFNAR1- (*n* = 4, green dots) treated mice as well as of PBS-treated control mice (*n* = 3, black dots) measured 6 days post infection. RNA values are expressed as copy number per ng of total RNA and the limit of detection is indicated as a dotted line. **(C, F)** Viral titers in the lungs (**C**) and brains (**F**) were determined 6 days after infection by median tissue culture infectious dose ($TCID_{50}$). **(D, G)** Representative immunohistochemical micrographs of lung (**D**) and brain (**G**) sections from PBS-treated control mice (left), isotype control- (middle) and α–IFNAR1- (right) treated mice at 6 days post infection. N-CoV-2 positive cells are depicted in brown. Scale bars, 1 mm. Right panels, quantification of the percentage of N-CoV-2 positive

area in the lung (top) and brain (bottom) of indicated mice; each dots represent one mouse.

Data are expressed as mean ± SEM. ** p-value < 0.01; Kruskal-Wallis test (**B-G**).

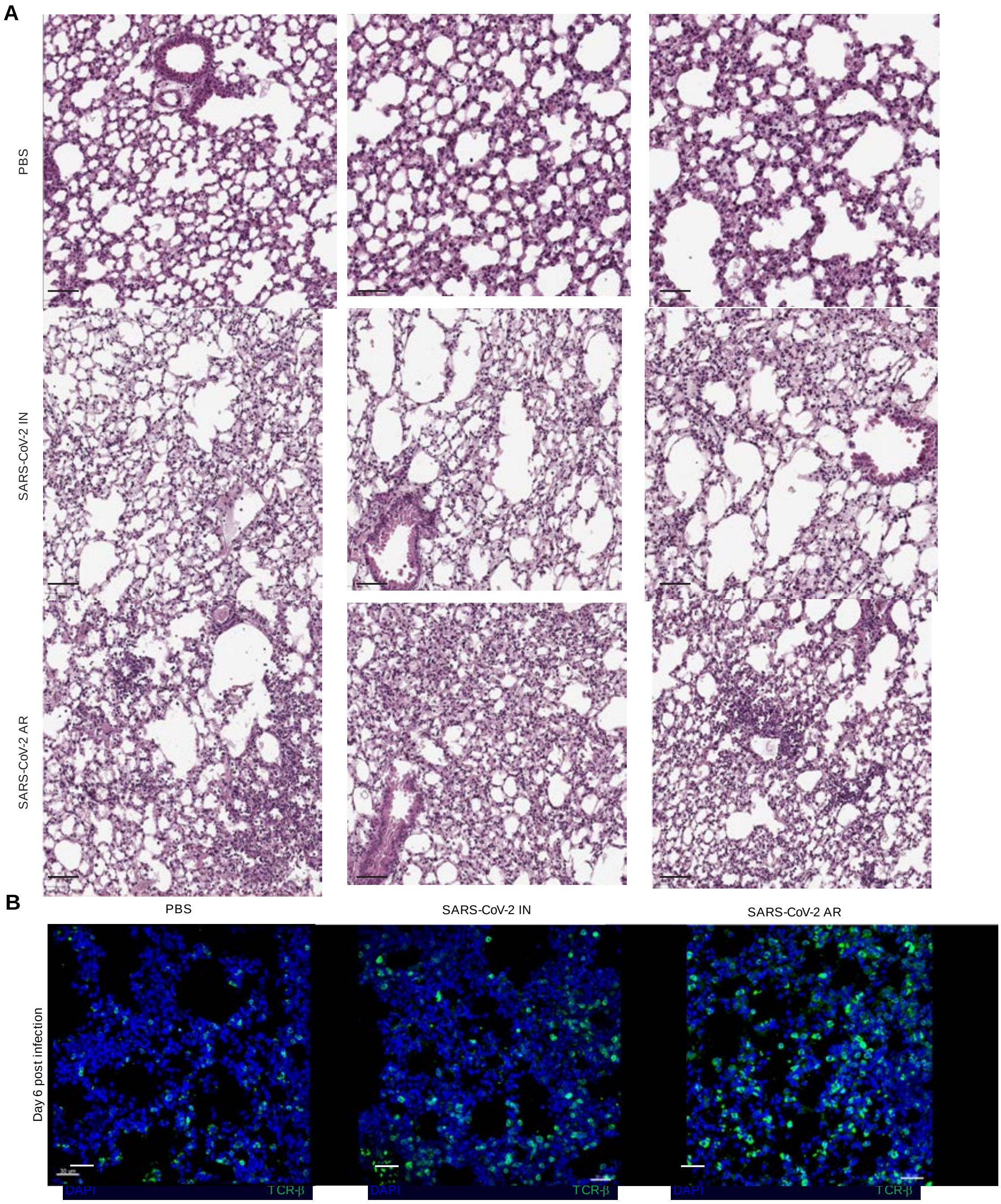

Figure S8. ***More severe lung pathology in AR-infected than in IN-infected mice.*** **(A)** Representative hematoxylin/eosin (H&E) micrographs of lung sections from PBS-treated control mice (top), IN- (middle) and AR-infected mice (bottom) 6 days post infection. Scale bars, 50 µm. **(B)** Representative confocal immunofluorescence micrographs of lung sections from PBS-treated control mice (left), IN- (middle) and AR-infected mice (right) 6 days post infection. TCR-β positive cells are depicted in green; cell nuclei are depicted in blue. Scale bars, 30 µm.

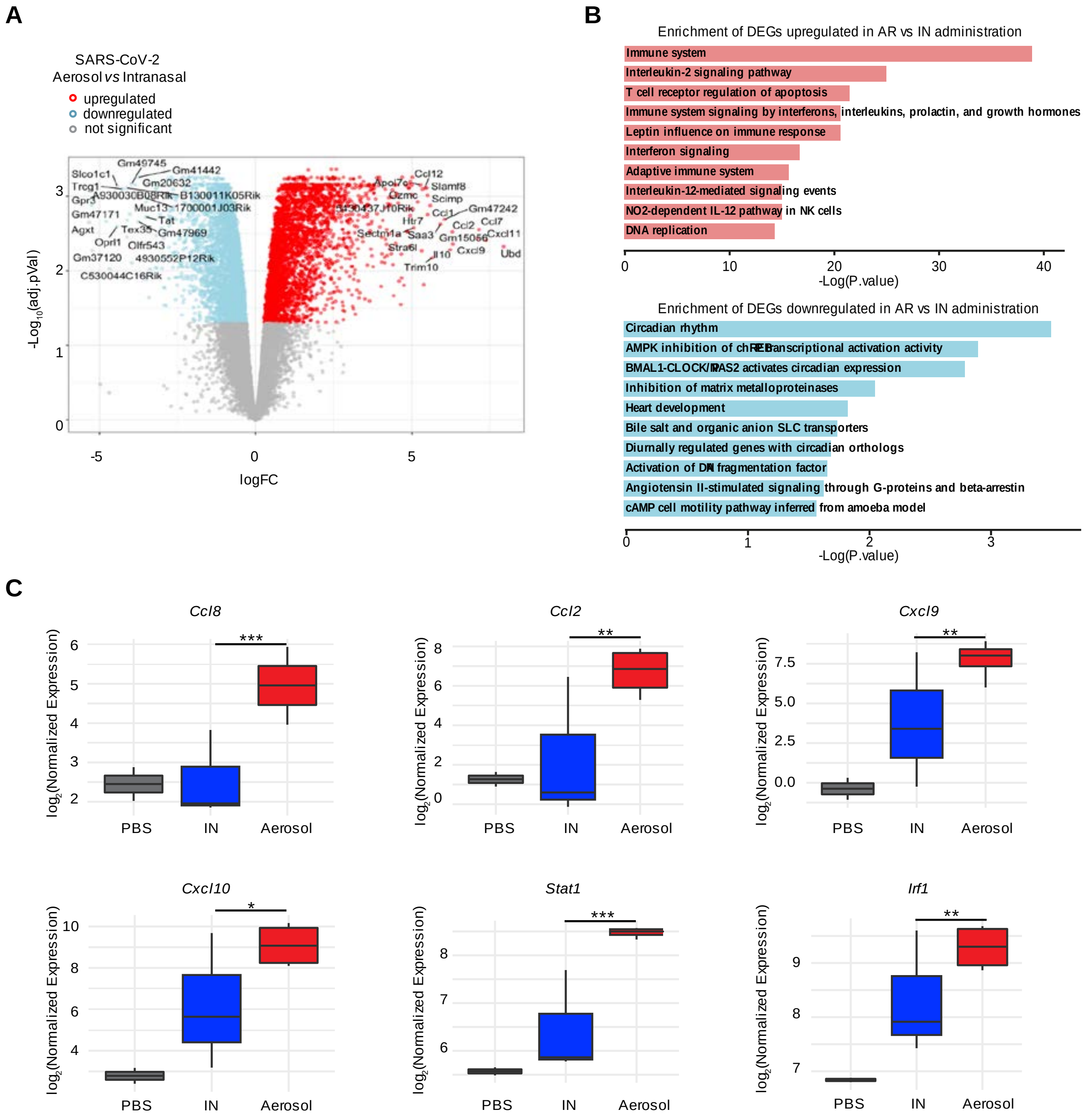

Figure S9. *Transcriptional signature in the lungs of infected mice.* (A) Volcano plot of RNA-seq results. The X-axis represents the Log2 Fold-Change of Differentially Expressed Genes (DEG) comparing AR- to IN-infected mice, the Y-axis the -Log10(FDR) Genes significantly upregulated in AR- relative to IN-infected mice (adjusted P value < 0.05) are colored in red and genes significantly downregulated are colored in blue. **(B)** Top ten pathways enriched by *p* values (resulting from a Fisher exact test) from BioPlanet 2019 database (Huang et al., 2019) for upregulated and downregulated genes (|logFC| > 1 and adjusted P value < 0.01). Enrichment tests were performed using the EnrichR web platform (https://maayanlab.cloud/Enrichr/). **(C)** Box plot representing the expression level of

the indicated genes in the lung of PBS-treated control mice (*n* = 2, gray boxes), IN- (*n* = 3, blue boxes) and AR-infected (*n* = 4, red boxes) mice 6 days post infection. Y-axis indicates the logarithmic normalized read counts. Comparison between IN-infection and AR-infection. * adjusted P value < 0.05, ** adjusted P value < 0.01, *** adjusted P value < 0.001. Adjusted P value corrected using Benjamini Hochberg correction method.

Supplementary Tables

Parameter	Degree	Scoring points
Fur	Shining	0
	Matte	1
	Ruffled	3
Posture	Normal	0
	Hunched	1
	Massively hunched	3
Activity level	Active (social contacts)	0
	Impaired (motility after stimulation)	1
	No activity (lethargy)	3
Eyes	Clear and clean	0
	Semi-closed	1
	Unclean and closed	3
Breathing	Normal	0
	Slightly changed	1
	Strongly accelerated	3

Table S1. *Clinical score to assess the severity of the disease in SARS-CoV-2-infected mice.* The clinical score was based on a cumulative 0-3 scale evaluating fur, posture, activity level, eyes and breathing.

Name	Clone	Source and catalog number	RRID
CD103	2E7	Biolegend #121408	AB_535950
CD62L	RM4-5	Biolegend #104420; Biolegend #104428	AB_493376; AB_830799
CD11b	M1/70	Biolegend #101218	AB_389327
IA/IE	M5/114.15.2	Biolegend #107622	AB_493727
Ly-6c	HK1.4	Biolegend #128013	AB_1732090
Siglec-F	E50-2440	BD Biosciences #740388	AB_2740118
CD64	X54-5/7.1	BD OptiBuild #740622	AB_2740319
CD8	53-6.7	Biolegend #100725; Biolegend #100759	AB_493425; AB_2563510
CD11c	HL3	BD Biosciences #563735	AB_2738394
CD4	RM4-5	BD Biosciences #740208; BD Biosciences #741912	AB_2734761; 2871226
B220	RA3-6B2	BD Biosciences #564662	AB_2722578
CD44	IM7	BD Biosciences #741227; BioLegend #103028	AB_2870781; AB_830785
CD69	H1.2F3	BD Biosciences #612793	AB_2870120
CD19	1D3	BD Biosciences #749027	AB_2873424
F4/80	BM8	Biolegend #123110	AB_893486
Ly-6g	1A5	BD Pharmingen #562700	AB_2737730
CD45	30-F11	Biolegend #103113; BD Biosciences #564279	AB_312978; AB_2651134
IFN-γ	XMG1.2	Biolegend #505813	AB_493312
Bcl-6	K112-91	Biolegend #581525	
TNF-α	MP6-XT22	Biolegend #506329	AB_11123912
CD183 (CXCR3)	CXCR3-173	BD Biosciences #740630	AB_2740325
CD279 (PD-1)	RMP1-30	BD Biosciences #749306	AB_2873680
Granzyme-B	GB12	Invitrogen #MHGB04	AB_10372671
T-bet	4B10	Invitrogen #25-5825-82	AB_11042699
CD3	145-2C11	BD Biosciences #564661	AB_2869596

Table S2. ***List of antibodies used for flow-cytometric analyses.***

Supplementary Data File Legends

Data file S1. ***Differential Gene Expression between AR- and IN-infected mice.*** Results showed in Figure 3F-I and Figure S9. Genes expressed (cpm ≥ 2 in at least 2 samples) were processed using LIMMA (Ritchie et al., 2015) and significant differentially expressed genes (Adjusted P value < 0.05) are showed.

Data file S2. ***Raw data file.***
